# Building a Health Literacy Indicator from Angola Demographic and Health Survey in 2015/2016

**DOI:** 10.3390/ijerph19052882

**Published:** 2022-03-01

**Authors:** Neida Neto Vicente Ramos, Inês Fronteira, Maria Rosário Oliveira Martins

**Affiliations:** Global Health and Tropical Medicine, Institute of Hygiene and Tropical Medicine, Nova University of Lisbon, 1249-008, Lisboa, Portugal; ifronteira@ihmt.unl.pt (I.F.); mrfom@ihmt.unl.pt (M.R.O.M.)

**Keywords:** health literacy, assessment tool, Angola, inequalities

## Abstract

Health literacy is a determinant factor for population health. It is important both for the prevention of health problems and the better management of those problems and unexpected situations that happen. Low health literacy has been consistently associated with poor health outcomes. This study aimed to develop a health literacy indicator for Angola and to analyze pertinent demographic characteristics related to it. Data were obtained from the first Angola Demographic and Health Survey conducted in 2015/16; we included 10 questions related to the American National Academy of Medicine definition of health literacy. Using factor analysis, we extracted one i indicator corresponding to four dimensions of health literacy. The indicator was dichotomized, and we used Logistic Regression to estimate factors associated with health literacy level: we obtained data from 19,785 adolescents and adults, aged 15–49 years. The internal consistency of the i indicator was reliable (Cronbach’s *α*  =  0.83). Adjusting for other variables, males with complete secondary education or above and living in urban areas were more likely to have a high level of health literacy. There were substantial differences between the 18 regions. This is the first study evaluating health literacy in Angola using the American National Academy of Medicine definition and a Demographic and Health survey. Our study shows unfavorable results for women, individuals living in rural areas and those less educated.

## 1. Introduction

The American National Academy of Medicine (ANAM) refers to health literacy as the degree to which individuals have the capacity to obtain, process, and understand basic health information and services needed to make appropriate health decisions [1,2]. Sorensen et al., 2012 [3] and Okan et al., 2019 [4] revisited the concept of health literacy, emphasizing the ability to access, understand, evaluate and apply health information to decision-making.

Poor health literacy can be viewed as a hidden risk factor as it is associated with many adverse health outcomes [5,6,7,8]. These include limited participation in cancer screening programs [9], increased hospital admissions, poor adherence to treatments and increase in late presenters at hospital emergency rooms that result in unfavorable outcomes [10]. Inadequate health literacy can contribute to reduced participation in prevention activities, higher prevalence of risk factors, worse self-management of chronic diseases [11] and poor disease outcomes [12,13], reduced effectiveness in communication with healthcare professionals [14], increased healthcare costs, worse functional status and poor overall health status [15]. Moreover, insufficient health literacy appears to be related with hesitancy towards vaccination against COVID-19 [16,17]; also, low literacy is associated with adherence to preventive measures due to a poor understanding of the symptoms of the disease, difficulty in being able to identify risk behaviors for infection, difficulty in finding reliable information and understanding government messages about COVID-19 [18,19]. The consequences of low levels of health literacy can be particularly severe in low or middle-lower income country (LMIC) contexts where few countries have accurately measured health literacy or its impact on health [20].

A systematic review, conducted in 2018, analyzed the status of tools for the assessment of health literacy from several perspectives and showed that most of the existing instruments are based on a multidimensional measurement of health literacy, mainly through abilities such as obtaining, understanding, and processing health-related information, as well as decision making [21]. This review also shows that the tools for measuring and monitoring health literacy from the 11 selected articles reviewed include nine countries, of which only one was from an African LMIC, i.e., Zambia [21]. 

Given the importance of measuring health literacy in LMICs particularly in Africa [15] and in the absence of reliable instruments, Demographic and Health Surveys (DHS) that produce high-quality data are important. Available in a consistent and coherent form, DHS represent a valuable opportunity to study the different dimensions of health literacy and are particularly useful when analyzing how health literacy levels vary within and across populations [22]. Recently, some authors have proposed an innovative way to construct a health literacy measure for sub-Saharan African countries based on DHS data [22,23,24]. Within this framework, health literacy is assessed by an indicator built using specific DHS questions related to the ANAM definition. The indicator, capturing four specific dimensions of health literacy, is derived using factor analysis. The procedure was first applied to Zambia using the 2015 DHS [22] and then to 13 other sub-Saharan African countries, using the most recent DHS data for each one [23]. However, the development and evaluation of reliable health literacy tools is still limited in Lusophone African countries that are left behind within this context.

This study aimed to develop and test the internal consistency and content validity of a health literacy indicator, based on data from the first DHS conducted for Angola (2015/2016) and to analyze sociodemographic characteristics related to health literacy levels. 

## 2. Materials and Methods

This is a cross-sectional study using secondary data collected from the first Demographic and Health survey conducted in Angola in 2015/16 [25].

Angola is an independent nation located in Southern Africa, where the official language is Portuguese [26,27]. The country has eighteen provinces with an estimated population of 29,310,273 inhabitants [27], of which 66% are under 25 years old. The human development index is 0.574 [28] and, according to UNESCO Institute for Statistics, the estimated general literacy level (based on the percentage of people that can read and write) of the adult population is 66% [26]. About 14.34% of the population use internet services [27]. 

Angola experienced 27 years of civil war, which weakened its government’s structure, education system, health system and services, leading to poor social indicators and an average life expectancy for the general population of around 62 years [28]. 

### 2.1. Study Sample

We selected information from 19,785 adults aged 15–49 years old who responded to the survey. Data were collected between October 2015 and March 2016. The survey was conducted in all Angola provinces in Portuguese [25]. The use and dissemination of the data was authorized by the Demographic and Health Surveys program [24]. DHS surveys in Angola are funded by the United States Agency for International Development (USAID) and administered by the National Institute of Statistics in Angola (INE) in collaboration with the Angolan Ministry of Health (MINSA).

### 2.2. Conceptualization and Construction of a Measure of Health Literacy for Angola

We construct a health literacy measure based on the four domains of the American National Academy of Medicine (ANAM) definition, which defines health literacy as the ability or capacity to interpret, obtain, understand and make appropriate health decisions [2]. Ten questions were identified within the Angola DHS that corresponded to the four domains of health literacy: interpretation skills were evaluated by questions about the aptitude to read a whole sentence or part of a sentence in the DHS; the capacity to obtain health information was evaluated by questions such as listening to the radio, reading a magazine or watching TV at least once per week; the capacity to understand the information obtained was measured by questions about obtaining family planning information through magazines, radio, TV, pamphlets and posters; and the capacity to make appropriate health decisions was assessed by questions concerning the knowledge of locations where a test for HIV/AIDS could be taken. 

Confirmatory factor analysis was used to identify the relationships between the variables and group them into a single factor that was derived from the ten survey questions. This factor represents the health literacy indicator. The internal consistency of the ten items was evaluated using Cronbach’s α. The ten items were summed after weighting each item by its factor loading, obtained using the simple oblique rotation method and Direct Oblimin with Kaiser normalization. The derived scores from the first factor, representing the 10 questions, is a continuous variable with zero mean and unit variance. This variable was transformed into tertiles and ordered from the lowest to the highest value; then, as suggested by several authors [22,23], we constructed the health literacy indicator as a two-category variable: high health literacy level (top tertile) and low health literacy level (bottom two tertiles). 

### 2.3. Validity

Validity of the health literacy measure was assessed as follows: We constructed an additional indicator to measure the degree of general knowledge about HIV/AIDS, defined by the sum of five questions related to general knowledge about HIV/AIDS contained in the DHS questionnaires. A variable indicating whether or not an individual had comprehensive knowledge of HIV/AIDS was then compared with the health literacy indicator, using an association measure [23]. This method has been previously used in the DHS, Millennium Development Goals [29], Sustainable Development Goals and two other studies [22,23].

### 2.4. Statistical Analysis

The demographic characteristics and health literacy items were summarized using descriptive statistics with weighted data [24]. 

A chi-squared test was used to analyze the association between health literacy levels and each demographic characteristic; we estimated unadjusted and adjusted logistic regression models to analyze factors associated with high health literacy levels. Variables included in the final model were chosen based on a literature review. Crude and adjusted odds ratios were computed together with corresponding 95% CI. We used the usual procedures to find outliers (box-plots), goodness-of-fit (Hosmer and Lemeshow test) and multi-collinearity (VIF) [30,31]. The level of significance was 5%.

### 2.5. Sensitivity Analysis

Our study used three different approaches to assess the sensitivity of the health literacy measure (dichotomous variable). First, we correlated the continuous scores obtained in factor analysis with the sociodemographic characteristics of the respondent using ordinary least squares (OLS) [23]. Second, we created a composite score in which each of the ten survey items were scored as one point if an affirmative response was given [23]. This continuous variable was then correlated with the respondent characteristics using OLS. Finally, we used another composite score in which media items were given a value of half a point, instead of one point. The continuous variable was correlated with the participant’s characteristics again using the OLS. Finally, we dichotomized continuous scores from the second and third approach using the median value. These dichotomized variables were associated with the participant’s characteristics using logistic regression [22,23]. We also created tertiles from these variables and dichotomized them on the lower tertile (lower two tertiles) corresponding to low health literacy and the top tertile (the higher tertile) corresponding to high health literacy. 

Statistical analysis was conducted with the software IBM Statistical Package for the Social Sciences 25.0 for Windows.

## 3. Results

We analyzed data from 19,785 individuals, of which 72% were women and 27% were men aged 15–49 years old. The profile of the participants was mostly urban (70.3%). Most of the individuals were young (69.6%). About 22.6% of the respondents have completed secondary education. Table 1 summarizes the general demographic characteristics of the sample. 

### 3.1. Deriving a Measure of Health Literacy by Factor Analysis

As can be seen in Table 2, to construct the health literacy measure, 10 questions from the demographic health survey related to the four dimensions of health literacy were used. The internal consistency was very good (Cronbach’s α = 0.83). The health literacy indicator comprises three factors (the eigenvalue was superior to 1), which accounted 67.66% of the total variance. The first factor is related to listening to the radio (loading = 0.64), watching TV (loading = 0.53), knowing a place to obtain a family planning method (loading = 0.51), and learning family planning information from the radio (loading = 0.79), TV (loading = 0.75) and magazines (loading = 0.30). The second factor is linked with the ability to read (loading = 0.92) and ability to write (loading = 0.93). The third factor includes the ability to learn family planning information from posters (loading = 0.88) and brochures (loading = 0.87). The health literacy indicator was obtained using only one factor through the simple oblique rotation method and Direct Oblimin with Kaiser normalization. This single factor, that combines the four dimensions of health literacy, accounts for 40.0% of the total variance. Participants categorized as having high health literacy responded affirmatively to an average of 7.4 of the 10 survey questions, while participants categorized as having low health literacy only responded affirmatively to an average of 3.1 of the 10 survey questions. As a whole, 31.4% had a high health literacy level and 68.6% had low health literacy. Disaggregating by gender, the prevalence of a high health literacy level is 44.9% for males and 27.2% for females.

### 3.2. Determinants of Health Literacy

**Figure 1:**
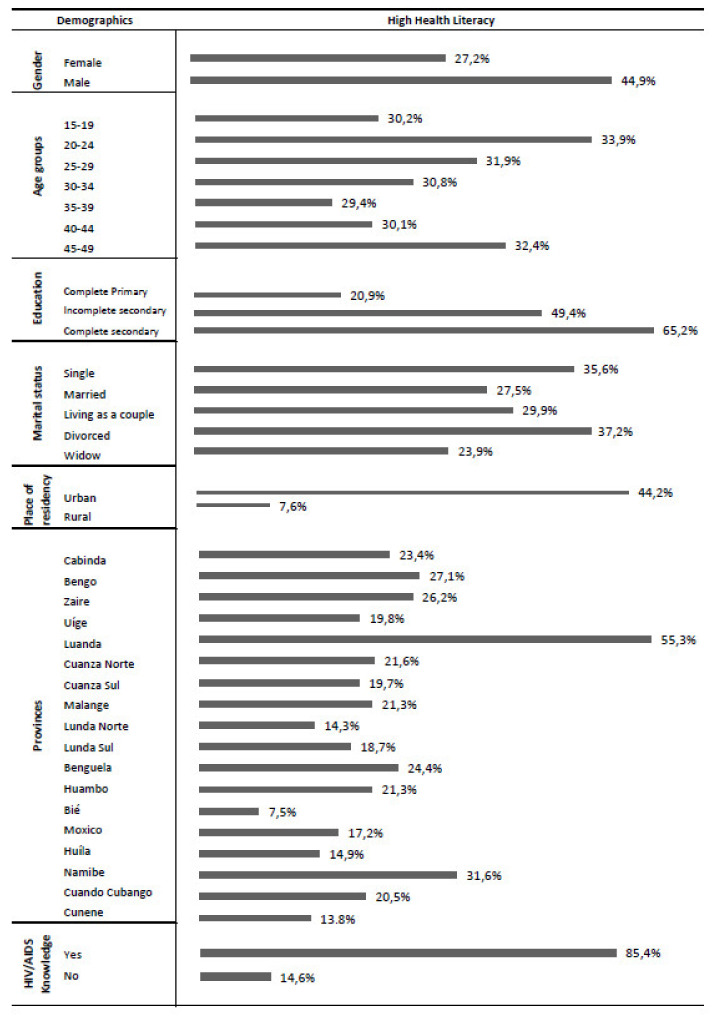
Frequency of high health literacy by socio-demographic characteristics.

As can be seen in Figure 1, the prevalence of high health literacy level was superior in males (44.9%), individuals with a complete secondary education (65%), unmarried individuals (52%) and in individuals living in urban areas (53%). Luanda, the capital, leads the ranking of the six provinces with the highest level of health literacy (53%) and individuals who have comprehensive knowledge about HIV/AIDS are those with greater levels of health literacy (85.4%).

Table 3 summarizes the results from the adjusted and unadjusted logistic regression models used to estimate the association between demographic variables and health literacy levels (classified as high or low). Adjusting for other variables, males with complete secondary education or above and living in urban areas were more likely to have a high level of health literacy. On the other hand, females with less than upper secondary education and living in rural areas were less likely to have a high level of health literacy.

### 3.3. Sensitivity Analysis

The alternative measure of health literacy obtained was a continuous variable and showed a similar correlation to respondent characteristics as the binary measure of health literacy when assessed in linear regression models. Results are not described here but can be made available by the authors. The multiple linear regression model was used as a complementary method to verify that the dependent variable, measured by health literacy (the scores that are a continuous variable), can be explained by the independent variables: gender, place of residence and highest level of education attended. The results show that the only coefficient beta that is not significantly different from zero (*p* value > 0.05) is the one related to marital status. 

## 4. Discussion

This is the first study assessing Angolan health literacy levels nationally. We applied factor analysis to construct a health literacy indicator, which has good properties (internal consistency and reliability) and was consistent with the health literacy concept defined by the ANAM [2]. This indicator has only two categories, high or low, and was built in accordance with the methodologies proposed by other authors that used DHS data for 14 African countries [23]. Unfortunately, no results were available in the literature for African Lusophone countries, such as Angola. 

**Figure 2:**
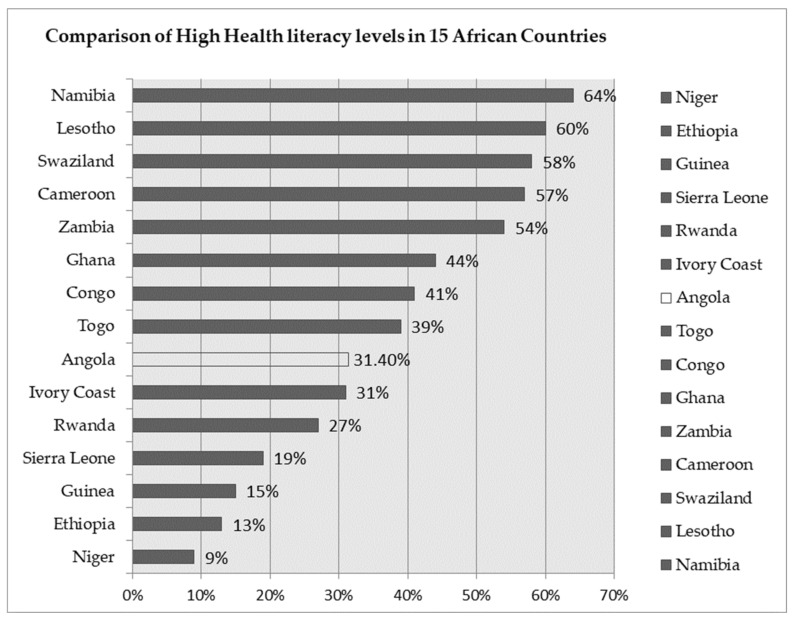
Comparison of high health literacy levels in 15 African countries. Source: Adapted from H. F. McClintock et al., 2019, with data from Angola.

We show that in Angola, 31.4% of the individuals had high health literacy levels, which reveals a low or inadequate level of health literacy for the Angola population. As can be seen in Figure 2, Angola is ranked at position 10 out of 15 African countries, with the prevalence of high health literacy below three of its neighboring countries, Congo (41%), Zambia (54%) and Namibia (64%) [23].

The high frequency of individuals with low levels of health literacy has revealed that health literacy deficits are a major challenge to global public health [4,15]. Although educational attainment is not an isolated factor in health literacy, the type of education system in which individuals are included plays a very important role. The average number of years of education for people aged 25 and above was 7.1 for Zambia, 6.9 for Namibia, 6.5 for Congo and 5.1 for Angola [32].

In our study, the prevalence of high health literacy in Angola differs considerably between men and women, with males showing the highest levels of health literacy (44.9%) and women only 27.2%. Even though this result is unsatisfactory for both groups, a significant inequality between gender is present and this result is consistent with those found for other African countries. In Egypt for example, the reported levels of health literacy were substantially lower for women when compared to men [33]. Additionally, for Zambia, the prevalence of a high health literacy level was 22% lower for women when compared with men [22]. Three studies conducted in Ghana showed a similar pattern: being a woman represented a significant predictor for low or limited health literacy [34,35,36]. 

In developing countries, particularly in sub-Saharan Africa, women generally have less economic power than men, low literacy, low digital literacy, low social status and limited access to media and health care [37,38,39]. Moreover, women in Angola, contrary to those in some more developed countries [40], are underrepresented in secondary or higher education; data from the Angolan DHS 2014/16 show this disparity: 43% of women aged 15–49 completed secondary or higher education levels compared with 63% of men. 

Our results also showed that the prevalence of high levels of health literacy was associated with a complete secondary education or higher levels of education. Individuals living in an urban context were more likely to have high levels of health literacy [41,42]. 

Health information is usually presented in a language that assumes that the individual has at least an eighth-grade education, though below this level, individuals are not able to fully understand what is presented or told to them about health [43,44,45].

In Angolan urban areas, where most individuals live, the prevalence of a high health literacy level was 44%. However, in rural areas, this prevalence only reached 7%. Luanda, the capital, leads the ranking of the six provinces with the top prevalence of a high health literacy level (53%), followed by Namibe (31.6%), Bengo (27.1%), Zaire (26.2%), Benguela (24.4%) and Cabinda (23.4%). This results must take into account the poverty index of the Angolan population [25,46], firstly because poverty is an important determinant of health literacy [8,37,38], and secondly, because the place where people live influences learning opportunities, the quality of schools, teacher certification and experience, and the development of adequate health literacy [47,48]. A systematic review on the differences between health literacy levels in urban and rural settings showed that rurality alone is not a risk factor for low literacy, yet when accompanied by the aforementioned factors and a health system that does not prioritize easy access to health care in rural areas, important disparities in health literacy may occur between urban, suburban and rural areas, particularly in developing countries [31,40,41]. However, comparisons have to be analyzed with caution; a recent meta-analysis has shown that when different assessment methods are applied to explore specific HL skills, the prevalence estimates of low health literacy differ significantly [13].

## 5. Limitations

Despite having used 10 questions from the DHS data directly related to the ANAM’s definition of health literacy, this study has some limitations [49]. 

The first is related to the limited number of items in the construction of the indicator as questions were taken from DHS database. Additionally, DHS data were not collected to assess health literacy. Consequently, our health literacy measure may have a lack of accuracy and applicability and should be applied just for this specific type of dataset. Another limitation is that we did not have the possibility to consider data from people aged 50 and over, for example.

Although this study was based on research methods applied in two previous studies [22,23] that aimed to assess health literacy in African countries, there may be some unintended Western perspective being applied to a non-Western culture, which could potentially lead to high bias.

## 6. Conclusions

Like the two previous studies published first for Zambia and then for 13 other sub-Saharan African countries that used the same methodology, our results contribute to improving the scarce scientific evidence on health literacy determinants in Africa, namely in a Lusophone context. 

The four domains of health literacy imply individually and collectively a set of capabilities to obtain, interpret and understand health information, as well as provide the foundation to make appropriate decisions at all levels of health prevention, i.e., controlling risk factors, treating diseases and rehabilitating people. This process is complex because it requires a systemic, multidisciplinary and intersectoral approach.

To our knowledge, this is the first study developing a robust health literacy indicator for Angola and is a unique population-based study on health literacy conducted in a Portuguese-speaking African country. Results show that there are inequalities in health literacy levels in Angola that deserve particular attention: women, rural residents and less-educated individuals. Potential public health targets could be directed at these subgroups. 

We hope that in the future, this type of health literacy measure can be used together with basic health indicators to produce impactful public health policies in order to change some health behaviors in Angola. 

## Figures and Tables

**Table 1:** Emographic characteristics.

Demographic Characteristics (n = 19,785)		
Variables	Categories	%
Gender	Female	71.6
	Male	27.2
Age groups	15–19	24.9
	20–24	20.6
	25–29	17
	45–49	6.6
Marital status	Not married	46.8
	Married	53.2
Residence	Urban	70.3
	Rural	29.7
Region	Cabinda	2.4
	Bengo	1.1
	Zaire	2.1
	Uíge	4.9
	Luanda	39.6
	Cuanza Norte	1.2
	Cuanza Sul	6.8
	Malange	3.1
	Lunda Norte	2.5
	Lunda Sul	1.6
	Benguela	8.1
	Huambo	6.4
	Bié	4
	Moxico	1.8
	Huíla	8
	Namibe	1.2
	Cuando Cubango	1.7
	Cunene	3.6
Level of Education	No education	0.3
	Incomplete primary	0.1
	Complete primary	40.6
	Incomplete secondary	29.6
	Complete secondary	22.6
	Higher Education Level	6.8

**Table 2:** Dimensions of health literacy and HIV/AIDS knowledge.

Variables	Categories	% of People Responding Yes
Health literacy	Primary school education	66.3
	Able to read whole sentence or part	63.1
	Read magazine at least once per week	37
	Listen to radio at least once per week	31.2
	Watch TV at least once per week	19.5
	Heard family planning information from magazine	13.2
	Heard family planning information from radio	28.3
	Heard family planning information from TV	29.9
	Heard family planning information from posters	13.2
	Heard family planning information from pamphlets	13.2
Knowledge of HIV/AIDS	Knows place to get an AIDS test	47.8
	Reduce chance of HIV by only having one sex partner without HIV	85.4
	Cannot get HIV from a mosquito bite	67.4
	Reduce chance of HIV by always using condoms correctly during sex	81.6
	People can get HIV if they share food with someone infected with HIV	69.6
	A healthy-looking person can have AIDS	75.1

**Table 3:** Factors associated with the probability of having a high health literacy level.

N = 19,738	Unadjusted High Health Literacy OR (IC 95%)	Adjusted High Health Literacy OR (IC 95%)
**Gender**		
Male	0.460 (0.426–0.497)	0.599 (0.545–0.659)
Female	ref	ref
**Age groups, years**		
15–19	0.903 (0.786–1.037)	0.414 (0.340–0.505)
20–24	1.071 (0.929–1.234)	0.625 (0.518–0.753)
25–29	0.978 (0.844–1.134)	0.677 (0.561–0.818)
30–34	0.929 (0.795–1.087)	0.797 (0.652–0.973)
35–39	0.871 (0.742–1.023)	0.907 (0.739–1.112)
40–44	0.899 (0.762–1.059)	0.879 (0.715–1.082)
45–49	ref	ref
**Education**		
≥Complete secondary and more	4.830 (4.459–5.231)	3.821 (3.491–4.181)
≤Complete primary	ref	ref
**Marital status**		
Married	1.204 (0.900–1.610)	0.910 (0.629–1.317)
Living as a couple	1358 (1.029–1.792)	1.108 (0.778–1.577)
Single	1.759 (1.332–2.323)	1.046 (0.725–1.507)
Separated	1.082 (0.795–1.474)	0.773 (0.525–1.138)
Divorced	1.947 (0.992–3.821)	1.012 (0.444–2.308)
widow	ref	ref
**Location of residence**		
Urban	9.717 (8.728–10.817)	5.316 (4.707–6.004)
Rural	ref	ref

## Data Availability

Data was obtained from DHS Program and are available at https://dhsprogram.com/data/dataset/Angola_Standard-DHS_2015.cfm?flag=0 with the permission of DHS.
